# Relationships between serotonin availability and frontolimbic response to fearful and threatening faces

**DOI:** 10.1038/s41598-023-28667-0

**Published:** 2023-01-27

**Authors:** R. Janet, N. Costes, I. Mérida, E. Derrington, J. C. Dreher

**Affiliations:** 1 CNRS-Institut de Sciences Cognitives Marc Jeannerod, UMR5229, Neuroeconomics, Reward, and Decision Making Laboratory, Lyon, France; 2 CERMEP-Imagerie du vivant, Lyon, France

**Keywords:** Cognitive neuroscience, Emotion, Social neuroscience

## Abstract

Serotonin is a critical neurotransmitter in the regulation of emotional behavior. Although emotion processing is known to engage a corticolimbic circuit, including the amygdala and prefrontal cortex, exactly how this brain system is modulated by serotonin remains unclear. Here, we hypothesized that serotonin modulates variability in excitability and functional connectivity within this circuit. We tested whether this modulation contributes to inter-individual differences in emotion processing. Using a multimodal neuroimaging approach with a simultaneous PET-3T fMRI scanner, we simultaneously acquired BOLD signal while participants viewed emotional faces depicting fear and anger, while also measuring serotonin transporter (SERT) levels, regulating serotonin functions. Individuals with higher activity of the medial amygdala BOLD in response to fearful or angry facial expressions, who were temperamentally more anxious, also exhibited lower SERT availability in the dorsal raphe nucleus (DRN). Moreover, higher connectivity of the medial amygdala with the left dorsolateral prefrontal and the anterior cingulate cortex was associated with lower levels of SERT availability in the DRN. These results demonstrate the association between the serotonin transporter level and emotion processing through changes in functional interactions between the amygdala and the prefrontal areas in healthy humans.

## Introduction

Serotonin (5-hydroxytryptamine or 5-HT) is a critical neurotransmitter in the generation and regulation of emotional behavior^1,2^. The serotoninergic neurons project from the raphe nuclei to the subcortical and cortical projection sites. Serotonergic neurotransmission has been an effective target for the pharmacological treatment of depressive and anxiety disorders^3^. Increasing evidence implicates serotonin signaling in the modulation of emotional behavior through its effects on a corticolimbic circuit that includes the amygdala and the prefrontal cortex^4,5^. Both of these structures are densely innervated by serotoninergic neurons and serotonin receptors are abundant throughout the amygdala sub-nuclei and prefrontal regions^5^.

The amygdala is of particular importance for processing facial expressions^4^, especially those depicting fear and anger^6,7^. This probably reflects the intrinsic survival value of threatening stimuli and information about danger in our environment. Amygdala serotonin synthesis correlates with the severity of social anxiety symptoms^8^ and was reduced, concomitantly, with stress-related amygdala activation, after successful pharmacological treatment^9^. The serotonin reuptake transporter (SERT) plays a key role in serotonergic neurotransmission by facilitating the reuptake of serotonin from the synaptic cleft and modulating serotonergic signaling by its central regulation. Recently, it has been argued that SERT in the DRN regulates serotonin release at the projection sites, with enhanced serotonin release in the synapse after inhibition by the selective serotonin reuptake inhibitors of the DRN^10^. In addition, a negative relationship between amygdala reactivity to facial emotions and SERT availability in the amygdala and/or raphe nucleus has been reported while participants were scanned in separate PET and fMRI sessions^11–14^. This established indirect evidence for a link between SERT availability and the brain circuit engaged in processing emotional facial expressions. However, a more direct relationship between the two signals, less dependent on external factors influencing their link is needed. The demonstration of a more direct link between dorsal raphe nucleus 5HT and the neural response to emotional facial expression requires a combination of SERT measure with BOLD signal recording in the same participants at the same time. Acquiring PET-fMRI data simultaneously ensures that participants are in the same emotional and cognitive states when the measurements are made, especially since mood, fatigue, and energy balance can all affect brain activity and serotoninergic transmission. Even if a multimodal paradigm can theoretically be performed using separated or non-simultaneous PET/MR systems^15,16^, the simultaneous acquisition allows the derivation of correlation maps that come from both modalities to describe the coupling of PET- and MR-derived parameters^17^.

Moreover, it should be noted that the amygdala is not a homogenous structure. For this reason, it is important to distinguish response properties of different amygdala subnuclei^18,19^. The amygdala can be divided into the medial and lateral parts on the basis of functional and connectivity analyses^20^. A greater density of SERT axons was demonstrated in the human medial amygdala compared to the lateral amygdala^21^, suggesting that these subregions convey different types of information. We therefore expected a stronger link between SERT level and amygdala BOLD signal in the medial amygdala. We also hypothesized that the SERT level may modulate connectivity between subregions of the amygdala and frontal regions.

Though mainly processed by the amygdala, facial emotions required a larger network to allow individuals to organize and flexibly regulate their emotional responses and integrate threat information to orchestrate a behavioral response. While abnormal amygdala reactivity to emotion is linked to emotional dysregulation^22,23^, perturbation of the connectivity between the amygdala and frontal regions may also participate in the dysregulation of negative emotion processing in clinical populations. We particularly focused on the connectivity between the amygdala and the dlPFC regions and the ACC, because both regions are involved in regulating emotions and the pattern of functional connectivity with the amygdala was reported to be perturbated in clinical populations. Functional interactions between the dlPFC and amygdala are critical for processing facial signals of anger and fear^24–27^. A recent meta-analytic study revealed a decrease in dorsolateral prefrontal activity during reappraisal in anxious individuals, suggesting dysregulation of this process^28^. Altogether, it appears that two distinct, but interplaying abnormalities, are combined in emotional dysregulation: abnormal amygdala emotional reactivity and abnormal regulation of this reactivity via prefrontal regions^29,30^. Similarly, the ACC, and particularly the pregenual ACC (pgACC), is part of a network involved in regulating emotions^31,32^. For example, a recent investigation revealed a decrease in functional connectivity between the pgACC and the amygdala in depressed individuals compared to healthy individuals^33^. The pgACC is believed to play a central role in the appraisal of emotions and serves as a hub to elaborate and conceptualize meaningful implications for self^34^. The pgACC plays an important role in the evaluation of emotional faces and receives dense projections from the amygdala^35^. The ventral ACC area, including the pgACC is a hub for emotional processes. Perturbation of the connectivity between the pgACC and amygdala may participate in the dysregulation of negative emotional face processing in clinical populations^36^. However, the relationship between the SERT level in the DRN and the connectivity between the amygdala and the pgACC remains unknown.

Focusing our analysis on fearful and angry facial expressions, that are biologically salient for the individual^37^, we tested whether increased SERT availability is linked to lower fronto-amygdala connectivity, especially with the dlPFC and pgACC. Using a new scanner that simultaneously records [^11^C]DASB PET and event-related 3T fMRI in the same participants, we investigated the link between SERT levels in the DRN and dlPFC/pgACC-amygdala connectivity directly. Our findings establish that the SERT level is linked to the dynamic interactions between the amygdala and the dlPFC/pgACC, which are critical for efficient emotional processing. Moreover, anxious traits were linked to amygdala reactivity to emotional faces.

## Methods

### Participants

Thirty-two healthy volunteers (only males; age range from 19 to 32 years; and mean age (M) 23.5 ± (SD) 3.01) were recruited through a mailing list from the University Claude Bernard Lyon 1. For inclusion in the study, participants were required to meet the following criteria: French-speaking, right-handed, no current medical treatment, no history of neurological or psychiatric disorders, and no auditory, olfactory or visual deficits. Furthermore, volunteers were screened for general MRI counter-indications. Two subjects were removed from the analysis resulting in a final sample size of thirty subjects. As a control for attention, participants had to answer a question at the end of each block by pressing a button box with their right index finger within a second. These two subjects were removed because of excessive failures to respond to the question presented at the end of each block suggesting that they did not pay attention to the stimuli. Among these two subjects, one explicitly told us in the debriefing that he did not look at the task. A physician conducted medical examinations concerning inclusion criteria such as physical and psychological health. Participants gave their written informed consent and received monetary compensation for the completion of the study. This study was approved by the Medical Ethics Committee (Comite de Protection des Personnes (CPP) Sud-Est IV, ID RCB: 2016-A01588-43) respecting the terms of the Declaration of Helsinki. All experiment and data analyses were subsequently carried out in accordance with the approved committee.

### General procedure

For all participants, the experiment took place in the afternoon. Participants were required to watch emotional faces using a pre-established Dynamic Facial Expression Task (a detailed description of the task is provided in the supplementary data, section Dynamic Facial Expression Task). The fMRI acquisition started in the last 20 min of the PET acquisition. After scanning, they completed scales assessing their state of anxiety and anxious traits: the two versions of the STAI (State-Trait-Anxiety-Inventory form Y), the YT (form Y-trait) version to assess anxious traits, and the YS (form Y-state) version to assess anxious states^38^. The injection of the radiotracer used in this study was performed before the beginning of the task and was followed by a resting state, allowing the tracer to reach its binding site^39^.

### PET data acquisition

PET and fMRI were performed simultaneously on a Siemens Biograph mMR. PET data were acquired in list-mode, over 90 min. The acquisition started with the intravenous injection of a bolus of [^11^C]DASB, a radiotracer that binds SERT. Mean [^11^C]DASB injected activity was 273.68 MBq, (SEM = 5.39). PET data were submitted to list mode motion correction^40^, then re-binned into 24-time frames (variable length frames, 8 × 15 s, 3 × 60 s, 5 × 120 s, 1 × 300 s, 7 × 600 s) for dynamic reconstruction. Images were reconstructed using OP-OSEM 3D incorporating the system point spread function using 3 iterations of 21 subsets. Sinograms were corrected for scatter, random, normalization, and attenuation^41^. Reconstructions were performed with a zoom of 2, which yielded a voxel size of 1.04 × 1.04 × 2.08 mm^3^ in a matrix of 344 × 344 × 127 voxels. Gaussian post-reconstruction filtering (FWHM = 2 mm) was applied to PET images.

### PET preprocessing and kinetic modeling

**Figure 1:**
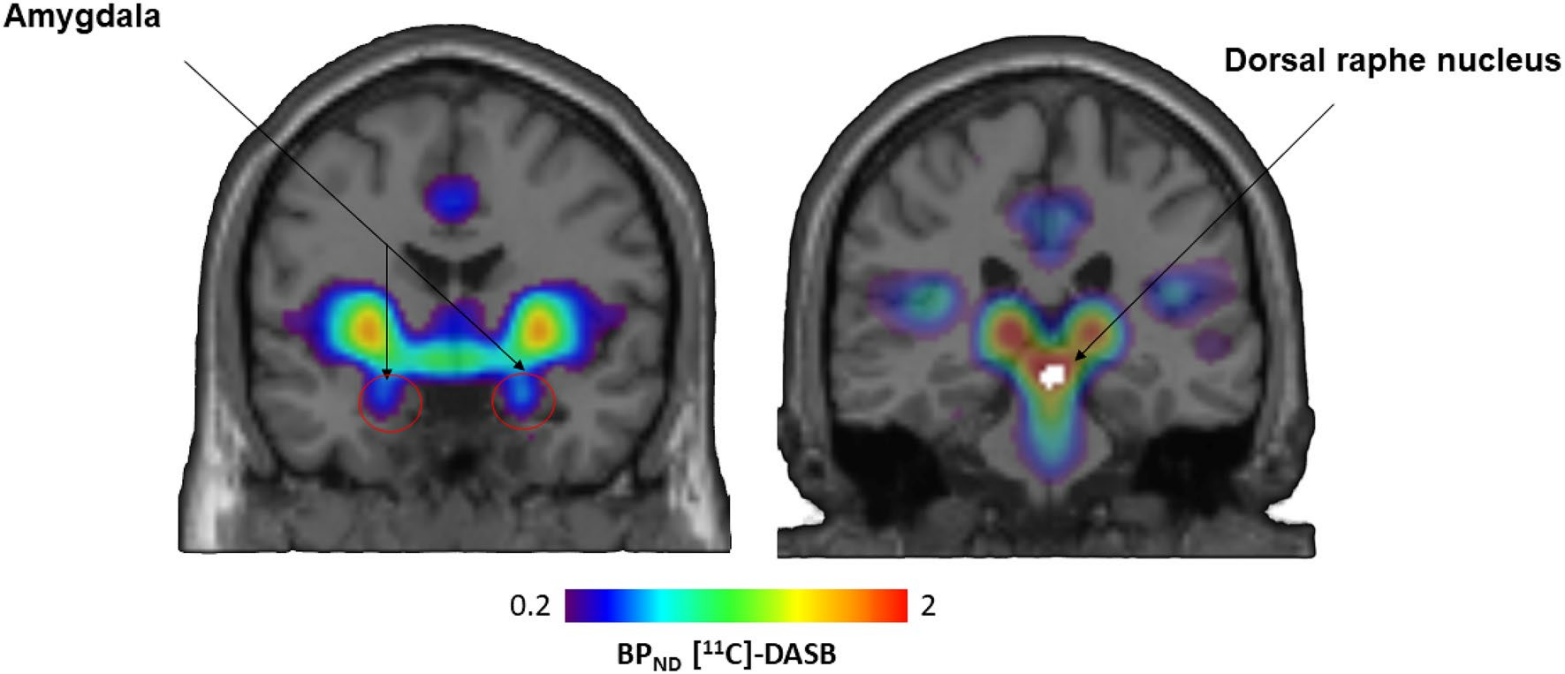
Statistical map of the BP_ND_ [^11^C]DASB showing SERT availability. The dorsal raphe nucleus (DRN) ROI used to assess SERT availability is shown in white.

For each participant, anatomical T1 MPRAGE was co-registered, (rigid transform), onto the average PET image. Regional labeling of brain structures was performed with the Hammersmith 83 Region Atlas^42,43^. This allowed to extract regional time-activity curves in the subject space, by wrapping the atlas from MNI space to the subject space. Parametric images of non-displaceable binding potential (BP_ND_) were computed by applying the Simplified Tissue Reference Model (SRTM)^44^, and using cerebellar grey matter, assumed to be devoid of SERT transporters, as a reference region^45^. BP_ND_, is defined as the ratio at the equilibrium of specific binding to non-displaceable uptake, reflecting the level of SERT availability (Fig. 1). PET parametric BP_ND_ images were spatially normalized into the standard Montreal Neurological Institute (MNI) atlas space using DARTEL toolbox procedure (The Diffeomorphic Anatomical Registration Through Exponentiated Lie Algebra)^46^. The resulting anatomical template was set to a voxel size of 1.5 × 1.5 × 1.5 mm.

### MRI data acquisition

All functional MRI acquisitions were performed using EPI BOLD sequences. Functional scans were performed using the following parameters: single-shot EPI, TR/TE = 2400/34, flip angle 85°, 52 axial slices interlaced 2 mm thickness 2 mm gap, FOV = 192 × 192 × 125. A total of 300 volumes were collected in an interleaved ascending manner. The first acquisition was performed after the stabilization of the signal.

Anatomical MRI acquisition consisted of 3D sagittal T1-weighted sequences, repetition time = 2300 ms; echo time = 2.34 ms; flip angle = 8; field of view = 256 mm; voxel size = 1 × 1 × 1 mm^3^. The anatomical volume covered the entire brain using 256 adjacent slices of 1-mm thickness.

### fMRI data preprocessing

Image analysis was performed using SPM12 (Wellcome Department of Imaging Neuroscience, Institute of Neurology, London, UK, https://www.fil.ion.ucl.ac.uk/spm/software/spm12/). Time-series images were registered in a 3D space to minimize any effect that could result from participant head-motion. Once DICOMs were imported, functional scans were corrected for slice timing, realigned to the first volume, and corrected for motion displacement and distortions (unwarping). First, we co-registered the anatomical images with the parametric PET images. Second, we used these co-registered anatomical scans as references and co-registered the functional MRI to them. Doing so, we ensured that the functional images are in the same space as the PET images. Finally, to perform group and individual comparisons, EPI Images were co-registered with structural maps, and normalized in the MNI space using the same DARTEL procedure as for parametric PET images, resulting in a voxel size of 1.5 × 1.5 × 1.5 mm. Images were then spatially smoothed with an 8 mm isotropic full-width at half-maximum (FWHM) Gaussian kernel using the standard procedures in SPM12. All functional images were inspected. We controlled for the motion between two interscan [Mean < 0.002, STD < 0.003, Max = 0.008, Min = − 0.006 for all x, y, and z translation and rotation]. According to these results, we did not need to exclude further participants or scans.

### Amygdala subregion reactivity to emotional faces

**Figure 2:**
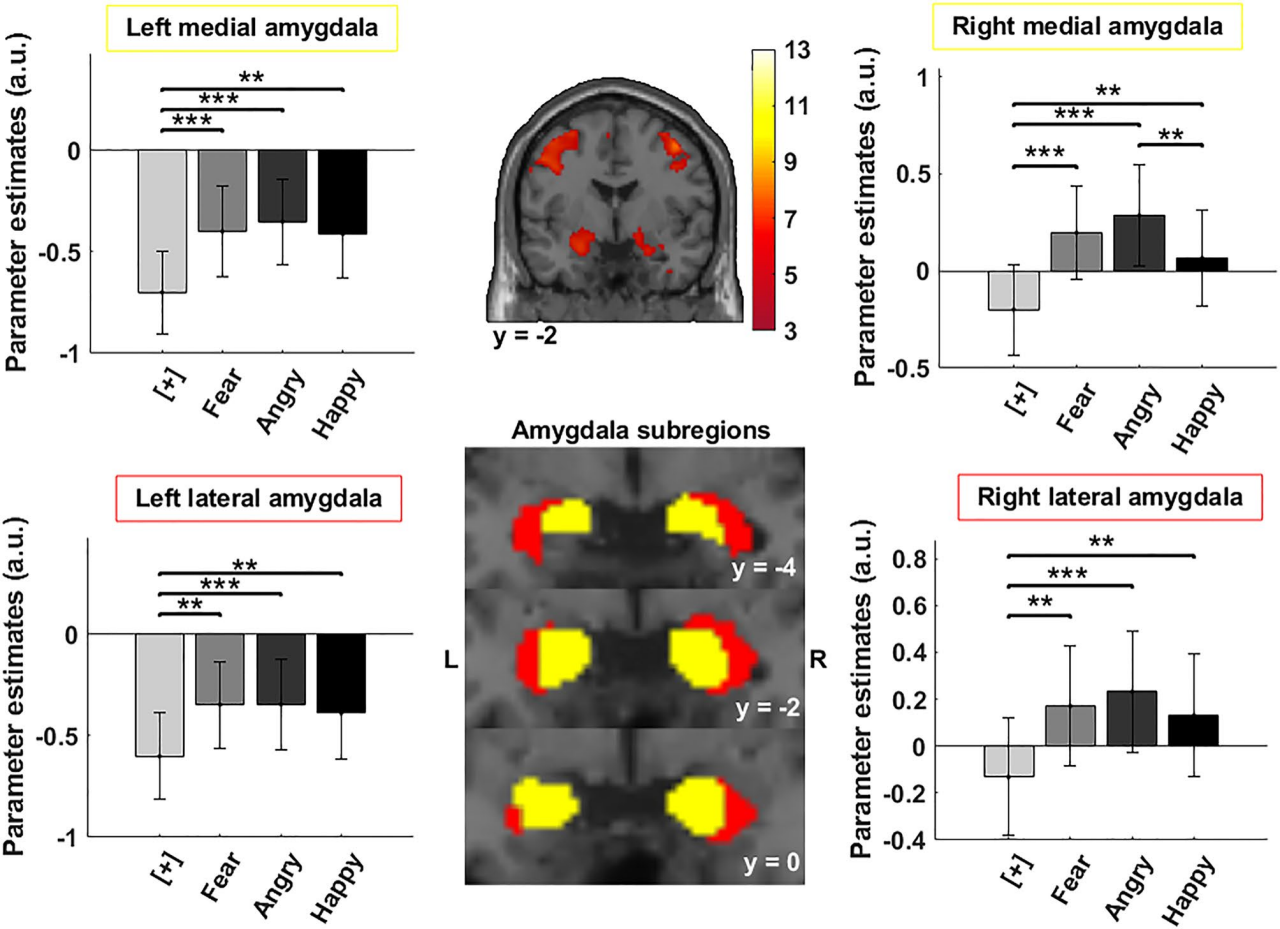
Amygdala subregional BOLD signal in response to emotional faces or fixation cross. Repeated measure ANOVAs were conducted to compare the sub-regional activation in the four conditions (fixation cross, Fear Face, Angry Face, and Happy Face), except for the right medial and the left lateral amygdala for which a Friedman test was conducted. These analyses showed that in all sub-nuclei, emotional faces significantly increased the BOLD signal compared to fixation (*p < 0.05, **p < 0.01 and ***p < 0.005; Bar graphs represent mean bold signal and error bars represent SEM).

To identify amygdala reactivity to emotional faces, we constructed a general linear model (GLM) including four regressors of interest denoting the Fixation Cross, Happy Face, Angry Face, and Fear Face conditions. This GLM also included a regressor representing the right index response given by the participant. For each participant, we created a contrast for each emotion condition and the fixation cross separately. Next, we entered the BOLD signal extracted from the four conditions, (Fixation and the three Emotion categories), in the four amygdala sub-regions (lateral and medial left and right amygdala) in a repeated measure ANOVA to determine if all regions from the amygdala responded to emotion conditions compared to the Fixation Cross condition. We also compared the contrast [Emotions > Fixation] created at the first level and ran a one-sample t-test at the group level to confirm that the amygdala was activated by emotions (Fig. 2).

### Whole-brain gPPI connectivity of amygdala related to negative emotion arousal.

We performed generalized psychophysiological interactions (gPPI) analysis to determine the connectivity of the amygdala subdivisions with other regions of the brain. To do this, we used the Connectivity Toolbox (Conn)^47^ and conducted a Seed-to-Voxel analysis. We loaded the preprocessed fMRI data into the toolbox and applied the de-noising step. This allowed us to de-noise the fMRI data by removing the signal relative to physiological parameters based on the CompCor method^48^. A high-pass filter (0.008 inf) was applied to the data. During the de-noising step, we added the motion parameters and their first derivative to eliminate the signal related to subject movements during data acquisition. We added the task events (our fixation cross and the three facial emotions) as confounds with no derivative. We also implemented SERT availability in the DRN as a covariate for the second-level analysis.

Finally, we performed four gPPI analyses, one for each of our amygdala ROIs, to observe covariance in connectivity between the fixation and negative emotions (fear and anger) conditions according to SERT availability in the DRN.

### ROI definition

Parametric images of BP_ND_ were used to extract the regional BP_ND_ of the [^11^C]DASB, mirroring the SERT availability.

The Dorsal Raphe Nucleus (DRN) has the highest concentration of serotonin neurons in the brain^45^. In addition, inhibition of the SERT DRN by selective reuptake inhibitors is known to enhance serotonin release in the terminalis^10^. Taking these results together, we reasoned that SERT DRN is an indicator of serotonin activity and thus we defined the DRN ROI using the Automated Anatomical Labelling (AAL3) atlas^49^ and computed its mean BP_ND_.

The amygdala is a key region involved in emotional processing and is known to be strongly engaged by our task, we therefore defined the amygdala as an ROI. Despite the fact that multiple sub-nuclei comprise the amygdala, it has been argued that the amygdala can be functionally divided into two main regions, the medial amygdala and the lateral amygdala^20^. Thus, we created two amygdala ROIs including the medial and lateral amygdala (for both the left and right amygdala) based on the functional subdivision provided by the Brainnetome Atlas. It allowed us to investigate the activity of the entire amygdala as well as the relative activity and connectivity of the sub-regions of the amygdala according to the level of SERT from the DRN.

Moreover, the anterior cingulate cortex is comprised of several regions^50^. In particular, the pregenual anterior cingulate cortex (pgACC) is known to participate in emotion processing, integration, and regulation^31,32^. A recent meta-analysis conducted by Marusak et al., in 2016 has shown that the ACC, and specifically the pgACC (x, y, z = 0, 40, 0) plays a key role in the development of emotional psychopathology^28^. To investigate the relationship between the SERT availability in the DRN and the amygdala-pgACC connectivity, we defined a sphere centered on the pgACC from the meta-analysis, with a radius of 10 mm. Concerning the dlPFC, this region plays a key role in the regulation of the emotional response. We used the term-based meta-analysis search function with the term “cognitive regulation” from the Neurosynth website. Using the resulting brain map, we identified two dlPFC regions, the left dlPFC (x, y, z = − 44, 22, 24), and the right dlPFC (x, y, z = 48, 18, 32). We thus defined a sphere, with a radius of 10 mm, centered on these two dlPFC peaks determined by the meta-analysis.

### Statistical analysis

All statistical analyses were performed using SPSS v21.0 (SPSS Inc., Chicago, IL, USA). First of all, normality was assessed using the Shapiro–Wilk test and histograms plots, and homoscedasticity using the Levene test. To compare BOLD signals for Fixation Cross, Fear, Angry, and Happy Face conditions, we used a repeated measure ANOVA with four factors except for the right medial and left lateral amygdala for which the normality assumption was violated. For the right medial and left lateral amygdala, a non-parametric Friedman test was performed. For multiple comparisons, post-hoc tests (with Bonferroni correction) were conducted. For multiple comparisons of the amygdala subnuclei activity, post-hoc tests (with Bonferroni correction) were conducted within each subnuclei for the emotion (leading to a statistical threshold of p < 0.0125). For correlations, the same normality assessment was carried out using the Shapiro–Wilk test and histograms plots. Then, according to the results of this test, either a Pearson (if the data were normally distributed) or a Spearman (if not) correlation test was performed. Correlations were considered significant if they passed the statistical threshold of p < 0.05.

## Results

### Statistical maps of SERT availability

The Binding Potential Non-Displaceable (BP_ND_) shows the availability of SERT. The map of the non-displaceable binding potential BP_ND_ revealed that the dorsal raphe nucleus (DRN), the region most enriched in serotoninergic neurons, is the region with the highest level of SERT (M = 2.24, SD = 0.38). We also observed a relatively high subcortical compared to the cortical concentration of SERT (M = 1.22 SD = 0.28 and M = 1.08 SD = 0.28, for the right and left ventral striatum respectively, M = 0.19, SD = 0.13 for the ventromedial prefrontal cortex). All these values were extracted from ROIs to illustrate this, (see supplementary data, section ROIs BP_ND_ extraction for detail). The mean BP_ND_ of the amygdala region was 0.34 with a standard error mean of 0.05 (Fig. 1).

### Amygdala and its sub-regional reactivity to emotional faces

The repeated measure ANOVA, conducted on the mean BOLD signal extracted from the ROIs, revealed that the medial region of the left amygdala and the lateral part of the right amygdala were significantly activated in the emotional face conditions compared to the Fixation Cross condition (F_(3,87)_ = 10.85; p < 0.001 for the left medial amygdala and F_(3,87)_ = 11.45; p < 0.001 for the right lateral amygdala). A Friedman test conducted for the right medial amygdala revealed a significant effect of the emotional conditions (χ^2^_(3)_ = 33.56, p < 0.001). A very similar effect of the emotion condition was revealed using a Friedman test for the left lateral amygdala (χ^2^_(3)_ = 21.4, p < 0.001). Post-hoc tests revealed that the BOLD signal significantly increased in all emotion conditions compared to the Fixation Cross condition (see Supplementary Table 1 for p-value and comparisons). It is notable that the right medial amygdala, but not the other regions of the amygdala, displayed significant differences in the Happy Face condition compared to the Angry Face condition (Fig. 2, Supplementary Table 1) (t_(29)_ = 2.91, p = 0.004). Note that applying a stronger correction accounting for the number of ROIs and Emotions, we do not find significant amygdala activity for Happy faces, nor any differences between the Happy faces and Angry faces (Supplementary Table 2).

### Dorsal raphe nucleus SERT availability correlates with BOLD signal related to emotions in the right amygdala

The first step was to reproduce previous findings obtained by studies that investigated the link between the amygdala BOLD signal and SERT level when these two modalities were acquired separately. To this end, we analyzed whether the BOLD signal related to the contrast [Emotions > Fixation] in the amygdala correlated with the SERT availability in the DRN. As predicted, the Pearson correlation revealed a significant negative correlation between the BOLD signal [Emotion-fixation] from the entire right amygdala and SERT availability in the DRN (p = 0.015, r = − 0.439). However, there was no significant correlation between the BOLD signal from the left amygdala as a whole and SERT availability in the DRN (p = 0.128). This result confirms, by simultaneous PET-fMRI acquisition, the previously observed link between amygdala activity and the SERT level. We also confirm that the observed results are not due to modulation of the cerebral blood flow by investigating the effect of the [^11^C]DASB clearance and variations in relative perfusion between target region (DRN) and reference region (cerebellum), see supplementary material.

Focusing with higher resolution on the distinct amygdala subregions, we investigated the correlations between the BOLD signal in the amygdala subregions and SERT availability in the DRN. Pearson correlation tests revealed a significant negative correlation between SERT availability in the DRN and BOLD signal in the right medial (p = 0.048, r = − 0.364) and lateral amygdala (p = 0.003, r = − 0.53). Such correlations were not found in the left medial (p = 0.114) or the left lateral amygdala (p = 0.669).

Negative emotions, including fear and anger, are biologically relevant for the individual, therefore we also tested whether the SERT level correlated with the BOLD signal evoked by such emotions in the right medial and lateral amygdala. Results revealed that SERT level in the DRN negatively correlates with the activity elicited by the Angry faces (p = 0.046, r = − 0.367) and Fear faces (p = 0.03, r = − 0.396) in the right medial amygdala but not in the right lateral amygdala (p = 0.075 and p = 0.077 for the Angry faces and Fear faces respectively). Similar negative correlations between activity and SERT level in the DRN were observed in the right medial amygdala (p = 0.006) and the right lateral amygdala (p = 0.046) when individuals were exposed to happy faces.

### Anxiety traits positively correlate with medial amygdala activity during emotion processing

**Figure 3:**
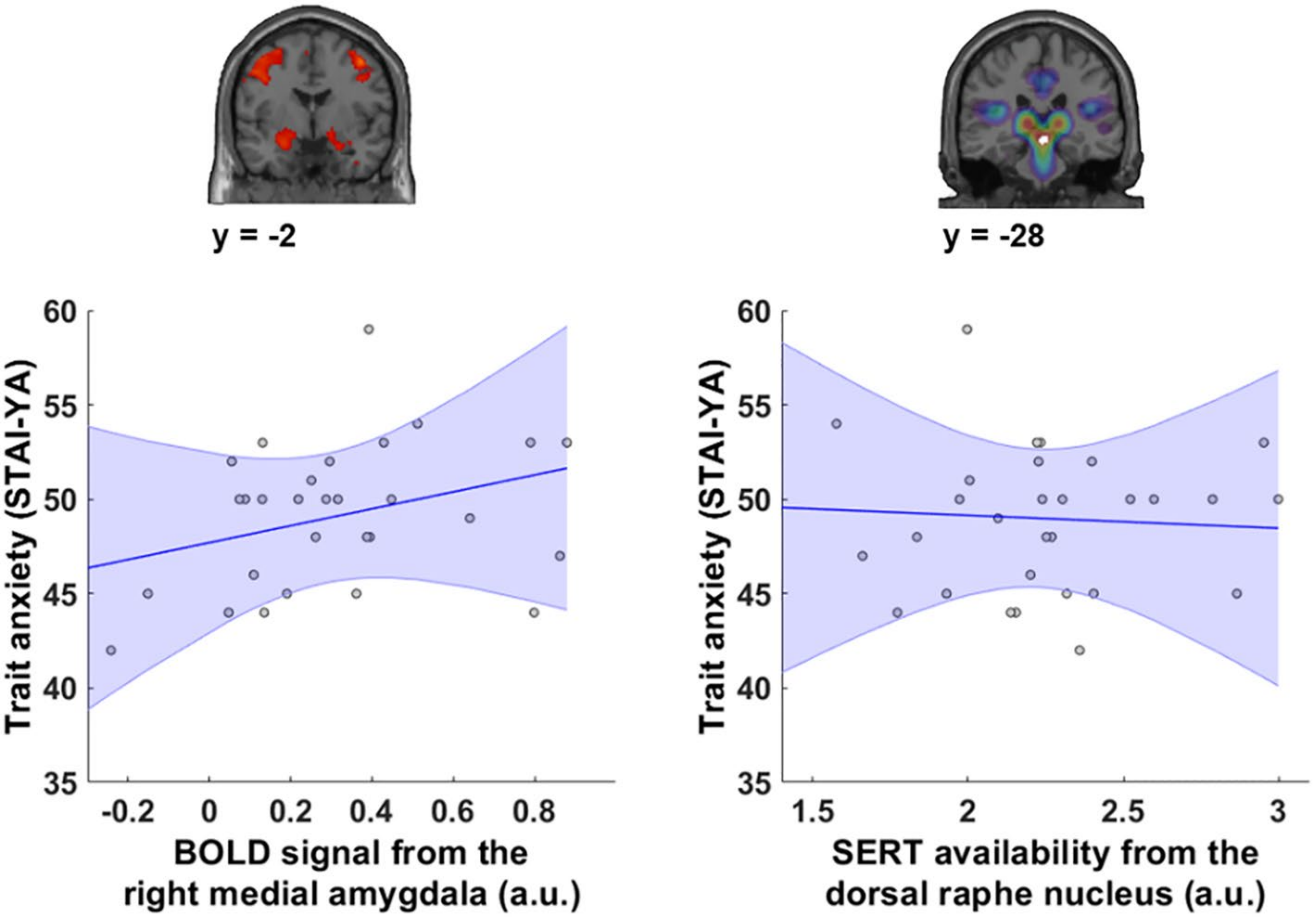
Correlation between anxious traits and right medial amygdala activity (left) and correlation between anxious traits and SERT availability in the dorsal raphe nucleus (right). A positive correlation is observed between the right medial amygdala activity (p < 0.05, r = 0.377) and anxious traits assessed using the STAI-YT. No correlation is observed between anxious traits and SERT availability in the dorsal raphe nucleus (p = 0.8).

We investigated the link between amygdala reactivity and anxious personality traits. A positive correlation between the score of the STAI-YT (trait) scale and reactivity to emotional conditions (all emotions included) was observed in the right medial part of the amygdala (r = 0.377, p = 0.040) (Fig. 3), but not in the lateral part of the right amygdala (p = 0.32). Excluding the happy faces stimuli and repeating the analysis, we found a similar relationship between trait anxiety and amygdala activity (r = 0.337, p = 0.034, one-tailed Pearson correlation). The same analyses were performed with the left medial and lateral amygdala and no significant correlations were observed (p = 0.588 and p = 0.705 for the left medial and lateral amygdala respectively). Finally, no link was observed between the level of SERT availability in the DRN and anxiety trait scores (p = 0.8) (Fig. 3).

### Effect of the DRN SERT availability on the functional connectivity of a prefronto-amygdala circuit when viewing negative emotions

**Figure 4:**
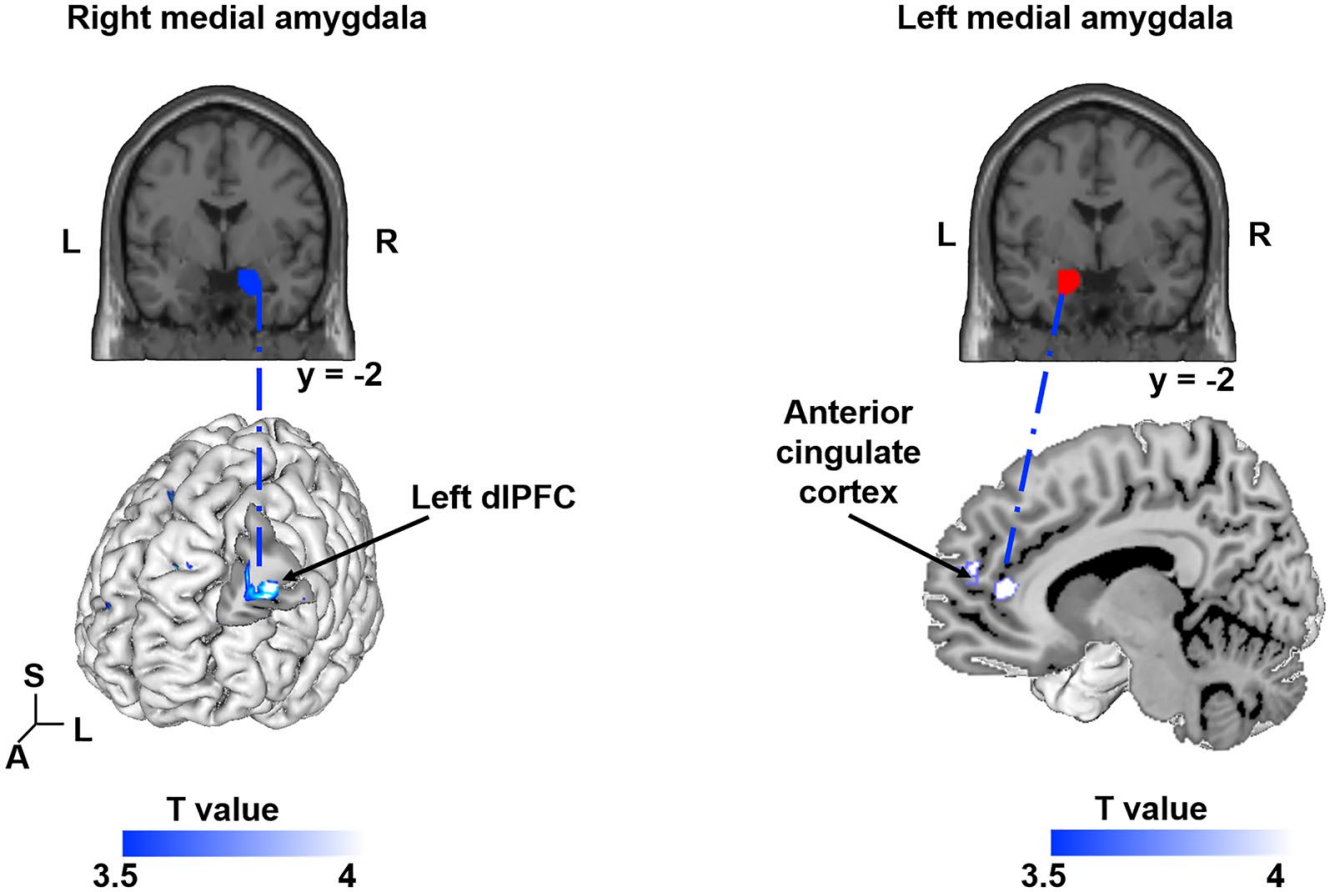
SERT availability in the dorsal raphe nucleus modulates the strength of the coupling between the medial amygdala and dorsolateral prefrontal/ACC when viewing negative emotional faces. A negative correlation was observed between the SERT level in the DRN and BOLD response in the comparison (Fear Face and Angry Face) > Fixation Cross. The connectivity between the right medial amygdala and the left dlPFC (x, y, z = − 40, 24, 26; t = 4.74) decreased as the SERT availability level in the DRN increased. (p < 0.05, family-wise error [FWE] small volume corrected in a sphere of radius of 10 mm centered on a meta-analysis peak x, y, z = − 44, 22, 24). In addition, results show that the connectivity between the left medial amygdala and the ACC (x, y, z = 8, 40, 6; t = 4.38) decreased as the SERT availability level in the DRN increased (p < 0.05, family-wise error [FWE] small volume corrected in a sphere of radius 10 mm centered on the meta-analysis peak x, y, z = 0, 40, 0).

We performed seed-to-voxel gPPI analyses to investigate the functional connectivity patterns of the amygdala subregions (medial vs lateral) that revealed significant activity in response to negative emotional faces stimuli. To do so, we analyzed the covariation of the connectivity differences between the Fixation Cross condition and the Fear and Anger Face conditions related to DRN SERT availability using the Conn Toolbox. We focused our attention on the negative emotion only because they exhibited greater amygdala activity in response to biologically salient stimuli (fearful or threatening facial expressions). The right medial amygdala showed a significant negative correlation between SERT availability in the DRN and its connectivity with the left dlPFC (P_SVC_ < 0.05, FWE corrected within the small volume sphere, with a radius of 10 mm, centered on previous meta-analysis activation x, y, z = − 44, 22, 24, peak activation x, y, z = − 40, 24, 26, t = 4.74), but not for the right dlPFC (Fig. 4). The gPPI analysis conducted with the left medial amygdala revealed a significant decrease of its functional connectivity with the anterior cingulate cortex (P_SVC_ < 0.05, FWE corrected within the small volume sphere centered on previous meta-analysis activation x, y, z = 0, 40, 0, with a radius of 10 mm, peak activation x, y, z = 8, 40, 6) (Fig. 4). All these results are summarized in Supplementary Table 3. No significant influence of SERT availability in the DRN was observed for the functional connectivity of either left or right lateral amygdala with other brain regions.

## Discussion

Using PET and fMRI simultaneously allowed us to investigate how inter-individual variations in SERT availability modulate brain activity engaged in processing fearful and angry faces. Our results indicate that individuals exhibiting higher BOLD response in the amygdala showed lower SERT availability while viewing emotional faces. Moreover, SERT availability in the DRN decreased the functional coupling between the right medial amygdala and the left dlPFC while viewing negative facial expressions. Furthermore, the right medial amygdala response to negative facial emotions was associated with increased anxious traits.

Our results revealed a negative correlation between the right amygdala activity and SERT availability in the DRN. This result, mirrors previous findings^13,14^ confirming that amygdala activity and the SERT level are linked. In a previous study, a negative relationship was observed between right amygdala BOLD fMRI activity and midbrain SERT^13^. In another study, a negative relationship was reported between amygdala reactivity and SERT in the amygdala, but not in DRN^12^. However, mood, fatigue, energy balance, and even circadian rhythm can all affect brain activity and the serotoninergic system^51,52^. Here, our simultaneous PET-fMRI acquisition ensures that participants were in the same emotional and cognitive states when the two measurements are made. The simultaneous acquisition also allows increased control of factors that could affect the link between the SERT level and the BOLD signal. Compared to these previous reports, which performed two separate acquisitions (one for the fMRI and one for the PET scans), our results are free from temporal inconsistencies between the two acquisitions. In addition, we investigated not only the relationship between the amygdala activity according to the SERT level in the DRN but how the connectivity of the amygdala is related to this SERT level.

We observed differences between left and right medial amygdala activation (cf Supplementary section Amygdala emotional reactivity lateralization). The right amygdala, especially the medial part, is less involved in processing positive emotions, because its response to happy faces was significantly different from its response to angry faces. This is consistent with the hypothesis that the amygdala is functionally asymmetric. It has been suggested that the right amygdala is involved in the rapid detection of emotional stimuli, whereas the left amygdala plays a role in the processing of more elaborate stimuli and sustained response evaluation^53,54^. The right amygdala may process emotional stimuli through a fast, automatic route, whereas the left amygdala processes emotional stimuli through a slower and regulated route^55–57^. This may explain why we observed significant differences in activation of the right amygdala in the Happy Face compared to the negative emotion face conditions, as this region is focused on rapid adaptation to stimuli, particularly those predicting negative situations. We can exclude that such differences are due to different levels of SERT in these regions because a direct comparison of the SERT levels in the right and left amygdala revealed no significant difference (p = 0.906).

The right amygdala activity correlated with anxious trait scores and was modulated by SERT availability in the dorsal raphe nucleus. This suggests that anxious traits are related to rapid, automatic processing of emotion and that the right amygdala reactivity towards facial emotions may be a neuronal marker of susceptibility to anxiety in the healthy population. No significant correlation was found between SERT availability in the DRN and anxious traits. This is in contrast with pathologically anxious individuals who have been shown to display an upregulation of SERT expression in the DRN, striatal and limbic regions^8,58^. Our results suggest that in healthy individuals, amygdala response is a better marker of anxious traits, whereas the overexpression of SERT contributes to pathological anxiety. Hyperreactivity of the amygdala to faces showing fear or anger expressions may result in inadequate treatment of the perception of internal states and environmental stimuli, potentially leading to anxiety and depression^59^. Many psychiatric disorders are associated with dysfunctional reactivity towards emotional faces, including anxiety disorders, depression and social phobia^60–62^. Furthermore, recent PET studies suggest an upregulated availability of SERT is associated with a pathological anxious state^9,58,63^. These findings strongly support a relationship between anxious traits in the healthy population and amygdala reactivity toward emotional stimuli. In addition, the similarity of the amygdala activity in all the amygdala subregions except the right medial part of the amygdala, for happy faces compared to fear and angry faces, can be explained by the fact that the amygdala is known to be engaged with increased salience (both positive and negative events)^37^.

Functional connectivity analyses revealed that in addition to its link with the amygdala activity, the SERT level is related to the coupling between the amygdala and left dlPFC and pgACC when individuals process negative emotions. The relative increase in SERT availability in the DRN was associated with reduced connectivity between the right medial amygdala and the dlPFC on one hand, and with reduced connectivity between the left medial amygdala and the pgACC, on the other. No similar functional connectivity effect was identified concerning the lateral amygdala. The medial part of the amygdala is more highly innervated by serotonergic neurons than the lateral part^21^. This difference in serotoninergic synapse density may explain the specific modulatory role of SERT availability in the functional circuitry engaging the medial amygdala. Amygdala-prefrontal connectivity has been associated with perseverative behavior and excessive worry in anxious individuals^36^, who also exhibited altered dlPFC activity^29,30^. When anxiety achieves pathological levels, this circuit is perturbated^29,30,36^. Disregulation of the SERT level could participate in the disruption of this functional coupling, contributing to pathological states. In turn, the dlPFC, which contributes to the appraisal of ongoing affective states triggered by subcortical regions, may regulate emotional responses to fearful/threatening stimuli in this circuit^24,26,64^. In rodents, the mPFC, a prefrontal area related to the human dlPFC, exerts an inhibitory influence on the amygdala outputs during fear conditioning^65,66^. This suggests that the SERT-modulated reduction in coupling between the dlPFC/pgACC and the medial amygdala that we observed in participants reflects that SERT shapes a functional feedback circuitry that regulates amygdala processing of environmental adversity. We did not include positive emotions in this gPPI analysis because negative events are more relevant to the literature on amygdala/serotonin and behaviors related to normal fear as well as pathological anxiety^67,68^. However, we also performed similar gPPI analysis using only the happy faces (c.f. Supplementary Data). Results revealed a significant negative correlation between SERT availability in the DRN and the connectivity with the ACC (x, y, z = 6, 36, − 4, t = 4.09) for the right medial amygdala only. Confirming previous reports showing connectivity between the amygdala sub-divisions and both positive and negative affect^69^ our results suggest that the amygdala activity and its connectivity with the ACC covaried with the SERT level in the DRN when viewing positive facial emotions.

Our findings clarify previous reports derived from genetic studies revealing that individuals with the short 5-HTTLPR showed either stronger coupling between the amygdala and the ventromedial prefrontal cortex^70^, or decreased coupling between the amygdala and the subgenual cortex^71^ during perceptual processing of fearful stimuli. Although these early studies provide indirect evidence of an association between genetic polymorphism of the SERT gene and functional interactions in the amygdala-frontal network involved in fear conditioning, there are many steps to establishing a link between these two measures (gene interactions, epigenetic variations, post transcriptional regulation, protein–protein interactions etc.). Our findings provide direct evidence by using a simultaneous multimodal neuroimaging approach that SERT availability modulates the functional coupling in the amygdala-frontal network. They support the idea that reduced availability of the SERT within the DRN, might model a relative reduction in gene function that is observed in individuals carrying the short variant of the 5-HTTLPR^72^. This would alter the network that mediates emotional reactivity via the amygdala, by reducing the functional connectivity between the amygdala and prefrontal regions.

Finally, our data also elucidates inter-individual differences in amygdala activity and SERT availability in the healthy population and possibly the reason for the variability in the treatment response in terms of efficacy and rapidity^73–75^. In fact, disrupted emotional processing present in anxiety and depression, a core clinical feature of these neuropathological disorders, is linked to SERT availability. SERT is the target of the Serotonin Selective Reuptake Inhibitors (SSRIs), commonly used to treat depression^76^. Treatment efficacy and rapidity differ greatly among individuals. Those who have higher SERT modulation by SSRI, respond faster and better^73–75^. It is reasonable to believe that individuals with low or high SERT availability in our study might develop such disorders during their life. However, they will probably experience differences in recovery. One possible reason for this is the different balance between amygdala activity and SERT availability, which does not offer the same flexibility after SSRI, affects the recovery process. Higher pretreatment diencephalic SERT availability is correlated with treatment response to SSRI 4 weeks later^77^. Increased susceptibility to depression and anxiety occurs/might be expected to occur in individuals with low SERT availability. A stronger fronto-amygdala coupling could, during stressful situations (i.e. facing an angry person), result in lower adaptability of the circuit and consequently a lower capacity to regulate emotional states.

Limitations. While confirming previous reports concerning a link between the amygdala activity and SERT levels in the DRN while individuals view emotional facial expression, we extend this to the functional connectivity between the amygdala and frontal regions. However, the results are correlational and further pharmacological approaches are required to confirm causality. Moreover, the present sample only includes males. Increasing the number of participants and adding females to the group would reinforce the observed results and also allow the investigation of possible gender differences.

Together, our multimodal neuroimaging approach established a direct role of SERT availability in regulating the amygdalo-frontal circuit which is crucial for integrating threatening stimuli. We also highlight inter-individual differences in the amygdalo-frontal network essential for effective emotion processing and clarify the link between SERT levels and functional coupling in this circuit. Understanding how between-subject variability in SERT levels influence brain activity and the circuitry at the system level may hold the key to a better understanding of associations between amygdalo-frontal functions, SERT and anxiety behavior.

## Supplementary Information

Supplementary Information.

## Data Availability

All data analyzed during the current study are available upon reasonable request to dreher@isc.cnrs.fr.

## References

[1] Raab K, Kirsch P, Mier D (2016). Understanding the impact of 5-HTTLPR, antidepressants, and acute tryptophan depletion on brain activation during facial emotion processing: A review of the imaging literature. Neurosci. Biobehav. Rev..

[2] Grady CL (2013). Acute pharmacologically induced shifts in serotonin availability abolish emotion-selective responses to negative face emotions in distinct brain networks. Eur. Neuropsychopharmacol..

[3] Mayo-Wilson E (2014). Psychological and pharmacological interventions for social anxiety disorder in adults: A systematic review and network meta-analysis. Lancet Psychiatry.

[4] LeDoux J (2003). The emotional brain, fear, and the amygdala. Cell Mol. Neurobiol..

[5] Savli M (2012). Normative database of the serotonergic system in healthy subjects using multi-tracer PET. Neuroimage.

[6] Davis M, Whalen PJ (2001). The amygdala: Vigilance and emotion. Mol. Psychiatry.

[7] Zald DH (2003). The human amygdala and the emotional evaluation of sensory stimuli. Brain Res. Rev..

[8] Frick A (2015). Serotonin synthesis and reuptake in social anxiety disorder a positron emission tomography study. JAMA Psychiat..

[9] Frick A (2016). Reduced serotonin synthesis and regional cerebral blood flow after anxiolytic treatment of social anxiety disorder. Eur. Neuropsychopharmacol..

[10] Dankoski EC, Carroll S, Wightman RM (2016). Acute selective serotonin reuptake inhibitors regulate the dorsal raphe nucleus causing amplification of terminal serotonin release. J. Neurochem..

[11] Ruhé HG (2014). Occupancy of serotonin transporters in the amygdala by paroxetine in association with attenuation of left amygdala activation by negative faces in major depressive disorder. Psychiatry Res. Neuroimaging.

[12] Rhodes RA (2007). Human 5-HT transporter availability predicts amygdala reactivity in vivo. J. Neurosci..

[13] Kobiella A (2011). How the serotonin transporter 5-HTTLPR polymorphism influences amygdala function: The roles of in vivo serotonin transporter expression and amygdala structure. Transl. Psychiatry.

[14] Schneck N (2015). Relationship of the serotonin transporter gene promoter polymorphism (5-HTTLPR) genotype and serotonin transporter binding to neural processing of negative emotional stimuli. J. Affect. Disord..

[15] Cavaliere, C. *et al.* Multimodal neuroimaging approach to variability of functional connectivity in disorders of consciousness: A PET/MRI pilot study. *Front. Neurol.* **9**, (2018). 10.3389/fneur.2018.00861 PMC6200912 30405513

[16] Karjalainen T (2017). Dissociable roles of cerebral μ-opioid and type 2 dopamine receptors in vicarious pain: A combined PET-fMRI study. Cereb. Cortex.

[17] Aiello, M., Cavaliere, C. & Salvatore, M. Hybrid PET/MR imaging and brain connectivity. *Front. Neurosci.* (2016). 10.3389/fnins.2016.00064 PMC4771762 26973446

[18] Ledoux, J. E. Emotion circuits in the brain. *Annu. Rev. Neurosci*. www.annualreviews.org (2000). 10.1146/annurev.neuro.23.1.155 10845062

[19] LeDoux J (2012). Rethinking the emotional brain. Neuron.

[20] Fan L (2016). The human brainnetome atlas: A new brain atlas based on connectional architecture. Cereb. Cortex.

[21] Lew CH (2019). Serotonergic innervation of the human amygdala and evolutionary implications. Am. J. Phys. Anthropol..

[22] Günther V (2020). Individual differences in anxiety and automatic amygdala response to fearful faces: A replication and extension of Etkin et al. (2004). Neuroimage Clin..

[23] Etkin A (2004). Individual differences in trait anxiety predict the response of the basolateral amygdala to unconsciously processed fearful faces. Neuron.

[24] Frank DW (2014). Emotion regulation: Quantitative meta-analysis of functional activation and deactivation. Neurosci. Biobehav. Rev..

[25] Hariri AR, Bookheimer SY, Mazziotta JC (2000). Modulating emotional responses: Effects of a neocortical network on the limbic system. NeuroReport.

[26] Lee H, Aaron SH, van Reekum CM, Brady N, Davidson RJ (2012). Amygdala-prefrontal coupling underlies individual differences in emotion regulation. Neuroimage.

[27] Ochsner KN, Gross JJ (2005). The cognitive control of emotion. Trends Cogn. Sci..

[28] Marusak HA (2016). You say ‘prefrontal cortex’ and I say ‘anterior cingulate’: Meta-analysis of spatial overlap in amygdala-to-prefrontal connectivity and internalizing symptomology. Transl. Psychiatry.

[29] Ball TM, Ramsawh HJ, Campbell-Sills L, Paulus MP, Stein MB (2015). Prefrontal dysfunction during emotion regulation in generalized anxiety and panic disorder. Psychol. Med..

[30] Robinson OJ (2015). Towards a mechanistic understanding of pathological anxiety: The dorsal medial prefrontal-amygdala ‘aversive amplification’ circuit in unmedicated generalized and social anxiety disorders. Lancet Psychiatry.

[31] Schiller D, Delgado MR (2010). Overlapping neural systems mediating extinction, reversal and regulation of fear. Trends Cogn. Sci..

[32] Etkin A, Egner T, Kalisch R (2011). Emotional processing in anterior cingulate and medial prefrontal cortex. Trends Cogn. Sci..

[33] Cheng W (2018). Functional connectivity of the human amygdala in health and in depression. Soc. Cogn. Affect. Neurosci..

[34] Dixon ML, Thiruchselvam R, Todd R, Christoff K (2017). Emotion and the prefrontal cortex: An integrative review challenges in understanding the role of the PFC in emotion. Psychol. Bull..

[35] Thomas P (2001). Primate anterior cingulate cortex: Where motor control drive and cognition interface. Nat. Rev. Neurosci..

[36] Makovac, E. *et al.* Alterations in amygdala-prefrontal functional connectivity account for excessive worry and autonomic dysregulation in generalized anxiety disorder. *Biol. Psychiatry* (2015). 10.1016/j.biopsych.2015.10.013 26682467

[37] Sander, D., Grafman, J. & Zalla, T. The human amygdala: An evolved system for relevance detection. *Rev. Neurosci*. **14** (2003). 10.1515/revneuro.2003.14.4.303 14640318

[38] Renzi DA (1985). State-trait anxiety inventory. Meas. Eval. Couns. Dev..

[39] Jae SK, Ichise M, Sangare J, Innis RB (2006). PET imaging of serotonin transporters with [11C]DASB: Test–retest reproducibility using a multilinear reference tissue parametric imaging method. J. Nucl. Med..

[40] Reilhac A (2018). Development of a dedicated rebinner with rigid motion correction for the mMR PET/MR scanner, and validation in a large cohort of 11C-PIB scans. J. Nucl. Med..

[41] Mérida I (2017). Multi-atlas attenuation correction supports full quantification of static and dynamic brain PET data in PET-MR. Phys. Med. Biol..

[42] Gousias IS (2008). Automatic segmentation of brain MRIs of 2-year-olds into 83 regions of interest. Neuroimage.

[43] Hammers A (2003). Three-dimensional maximum probability atlas of the human brain, with particular reference to the temporal lobe. Hum. Brain Mapp..

[44] Lammertsma AA, Hume SP (1996). Simplified reference tissue model for PET receptor studies. Neuroimage.

[45] Kish SJ (2005). Regional distribution of serotonin transporter protein in postmortem human brain: Is the cerebellum a SERT-free brain region?. Nucl. Med. Biol..

[46] Ashburner J (2007). A fast diffeomorphic image registration algorithm. Neuroimage.

[47] Whitfield-Gabrieli S, Nieto-Castanon A (2012). Conn: A functional connectivity toolbox for correlated and anticorrelated brain networks. Brain Connect..

[48] Behzadi Y (2007). A component based noise correction method (CompCor) for BOLD and perfusion based fMRI. Neuroimage.

[49] Rolls ET, Huang CC, Lin CP, Feng J, Joliot M (2020). Automated anatomical labelling atlas 3. Neuroimage.

[50] Neubert Franz-Xaver, Mars Rogier B., Sallet Jérôme, Rushworth Matthew F. S. (2015). Connectivity reveals relationship of brain areas for reward-guided learning and decision making in human and monkey frontal cortex. Proceedings of the National Academy of Sciences.

[51] Markus CR (2008). Dietary amino acids and brain serotonin function; Implications for stress-related affective changes. NeuroMol. Med..

[52] Daut, R. A. & Fonken, L. K. Circadian regulation of depression: A role for serotonin. *Front. Neuroendocrinol.* (2019). 10.1016/j.yfrne.2019.04.003 PMC9826732 31002895

[53] Baas D, Aleman A, Kahn RS (2004). Lateralization of amygdala activation: A systematic review of functional neuroimaging studies. Brain Res. Rev..

[54] Kilpatrick L, Cahill L (2003). Amygdala modulation of parahippocampal and frontal regions during emotionally influenced memory storage. Neuroimage.

[55] Gläscher J, Adolphs R (2003). Processing of the arousal of subliminal and supraliminal emotional stimuli by the human amygdala. J. Neurosci..

[56] Hung Y (2010). Unattended emotional faces elicit early lateralized amygdala-frontal and fusiform activations. Neuroimage.

[57] Wright CI (2001). Differential prefrontal cortex and amygdala habituation to repeatedly presented emotional stimuli. NeuroReport.

[58] Hjorth, O. R. *et al.* Expression and co-expression of serotonin and dopamine transporters in social anxiety disorder: A multitracer positron emission tomography study. *Mol. Psychiatry* (2019). 10.1038/s41380-019-0618-7 31822819

[59] Canli T, Lesch K-P (2007). Long story short: The serotonin transporter in emotion regulation and social cognition. Nat. Neurosci..

[60] Demenescu LR (2013). Amygdala activation and its functional connectivity during perception of emotional faces in social phobia and panic disorder. J. Psychiatr. Res..

[61] Harmer CJ, Cowen PJ (2013). ‘It’s the way that you look at it’-a cognitive neuropsychological account of SSRI action in depression. Philos. Trans. R. Soc. B. Biol. Sci..

[62] Stein M (2007). Increased amygdala and insula activation during emotion processing in anxiety-prone subjects. Am. J. Psychiatry.

[63] Iscan Z (2018). A positron emission tomography study of the serotonergic system in relation to anxiety in depression. Physiol. Behav..

[64] Yang M, Tsai SJ, Li CSR (2020). Concurrent amygdalar and ventromedial prefrontal cortical responses during emotion processing: A meta-analysis of the effects of valence of emotion and passive exposure versus active regulation. Brain Struct. Funct..

[65] Moustafa AA (2013). A model of amygdala-hippocampal-prefrontal interaction in fear conditioning and extinction in animals. Brain Cogn..

[66] Sotres-Bayon F, Bush DEA, LeDoux JE (2004). Emotional perseveration: An update on prefrontal-amygdala interactions in fear extinction. Learn. Mem..

[67] Hariri AR, Tessitore A, Mattay VS, Fera F, Weinberger DR (2002). The amygdala response to emotional stimuli: A comparison of faces and scenes. Neuroimage.

[68] Hariri AR (2002). Serotonin transporter genetic variation and the response of the human amygdala. Science.

[69] Klein-Flügge, M. C. *et al.* Relationship between nuclei-specific amygdala connectivity and mental health dimensions in humans. *Nat. Hum. Behav.* (2022). 10.1038/s41562-022-01434-3 PMC7613949 36138220

[70] Heinz A (2005). Amygdala-prefrontal coupling depends on a genetic variation of the serotonin transporter. Nat. Neurosci..

[71] Pezawas L (2005). 5-HTTLPR polymorphism impacts human cingulate-amygdala interactions: A genetic susceptibility mechanism for depression. Nat. Neurosci..

[72] Lesch K-P (1996). Association of anxiety-related traits with a polymorphism in the serotonin transporter gene regulatory region. Science.

[73] Keers R (2011). Interaction between serotonin transporter gene variants and life events predicts response to antidepressants in the GENDEP project. Pharmacogenom. J..

[74] Porcelli S, Fabbri C, Serretti A (2012). Meta-analysis of serotonin transporter gene promoter polymorphism (5-HTTLPR) association with antidepressant efficacy. Eur. Neuropsychopharmacol..

[75] Ruhé HG (2009). Serotonin transporter gene promoter polymorphisms modify the association between paroxetine serotonin transporter occupancy and clinical response in major depressive disorder. Pharmacogenet. Genom..

[76] Vaswani M, Linda FK, Ramesh S (2003). Role of selective serotonin reuptake inhibitors in psychiatric disorders: A comprehensive review. Prog. Neuropsychopharmacol. Biol. Psychiatry.

[77] Kugaya A (2004). Brain serotonin transporter availability predicts treatment response to selective serotonin reuptake inhibitors. Biol. Psychiatry.

