# Supplementary material for: Relationships between serotonin availability and frontolimbic response to fearful and threatening faces
Source: Sci Rep. 2023 Jan 27;13:1558. doi: 10.1038/s41598-023-28667-0 (PMC9883493; doi:10.1038/s41598-023-28667-0)
Supplement: Supplementary file 1 — Supplementary Information. [file 41598_2023_28667_MOESM1_ESM.docx]

## **Supplementary materials**

**Supplementary methods**

**Dynamic Facial Expression Task**

Amygdala is a key region for the perceptual processing of emotional faces. This is even more robust for dynamic processing of faces (movies) rather than static faces ^1^. Participants passively viewed blocks of faces morphing dynamically into either an angry, fearful, or happy facial expression (Angry Face, Fear Face, and Happy Face conditions). All these face stimuli came from the valid and reliable pictures of facial affect ^2^. The control baseline condition was the Fixation Cross condition. Stimuli consisted of short 133-msec animation clips for each of 10 different faces, showing a morphing sequence consisting of four frames (55%, 70%, 85%, and 100% emotional expression) repeated at 2 Hz. An experimental session lasted 11.55 min and consisted of six blocks of each emotion (25 sec, 50 morphing sequences each) and nine blocks of fixation cross (25 sec, baseline for analysis) (**supplementary figure 1**). The order of blocks was pseudo-randomized; adjacent blocks of the same emotion or fixation cross were avoided. A Fixation Cross block started and ended each session. As a control for attention, participants had to answer a question at the end of each block by pressing on a button box with their right index finger within a second. Due to excessive failures to respond, two subjects were removed. Post scan interviews confirmed that they had not paid attention to the faces. Stimuli were presented, and the responses to the stimuli were collected using the Psychotoolbox Toolbox Version 3 (PTB-3) on a MATLAB (2013a) version 7.16.0 (R2013a). Natick, Massachusetts: The MathWorks Inc.

**ROIs BP_ND_ extraction**

For completeness, we also used the nucleus accumbens and a cortical region, the ventromedial prefrontal cortex, to extract the Binding Potential of the [^11^C] DASB level within this region and illustrate the SERT distribution (**supplementary figure S2**). The ventromedial prefrontal cortex was defined using a sphere ROI, with a 4 mm radius and centered on the following coordinates x:-4, y:52, z:-3. These ROIs were created for illustration purposes of the SERT distribution. The nucleus accumbens was defined using the AAL3 atlas.

**gPPI connectivity of the sub-regional amygdala for the happy emotion**

For completeness, we performed similar gPPI analyses to look at differences in connectivity between the fixation cross and all the happy face stimuli according to SERT availability in the DRN for each of our amygdala ROIs that had revealed significant BOLD signal changes in response to negative facial emotions. To do so, we analyzed the covariation of the connectivity differences between the Fixation Cross and the Happy Face condition related to the dorsal raphe SERT availability, using the Conn Toolbox. Here again, we applied small volume correction to the ROIs of interest defined in the method section.

**Supplementary results**

**Amygdala emotional reactivity lateralization**

To investigate lateralization, we conducted a repeated measure ANOVA, including three different factors. The first factor denoted the lateralization, with two levels (right and left amygdala). The second factor denoted the sub-nuclei of the amygdala (medial or lateral). The last factor denoted the Emotion condition (Fear, Angry, Happy and fixation cross). Results revealed a significant main effect of the Emotion condition (F_(3,87)_ = 18.45; p < 0.001). There was also a significant main effect of the lateralization (F_(1,29)_ = 5.02; p = 0.033). This confirmed the effect of emotion compared to fixation cross on amygdala activity and suggests that there are differences in this activity according to the lateralization. It also revealed a significant interaction effect between the emotion conditions and the sub-nuclei of the amygdala (F(3,87) = 3.42: p = 0.021). No other significant effects were observed.

**Partial correlation analyses between SERT availability and amygdala BOLD response**

Because SERT availability levels in the amygdala were highly correlated with those in the DRN (p < 0.001, r = 0.77 and r = 0.78 for left and right amygdala respectively), we conducted partial correlation tests. A first partial correlation was conducted between SERT availability and the BOLD signal from the right amygdala, controlling for SERT availability in the DRN. No significant correlation was observed. However, partial correlation analysis between the BOLD signal from the right amygdala and SERT availability in the DRN, controlling for SERT availability in the right amygdala, revealed a significant negative correlation between the BOLD signal from the right amygdala and SERT availability in the DRN (p = 0.014, r = -0.452). This shows that the right amygdala reactivity to emotional faces is related to SERT availability in the DRN more than to the local SERT availability in the right amygdala. Thus, there is a stronger link between the amygdala reactivity and the dorsal raphe SERT availability than with the amygdala SERT availability. This also comforts our choice of using the SERT level from the DRN for our main analysis.

The same partial correlation procedures were performed in the left amygdala. No significant correlations were found between the BOLD signal and SERT availability in the left amygdala (p = 0.139) when controlling for SERT availability in the DRN, nor between the BOLD signal from the left amygdala and SERT availability in the DRN (p = 0.829) when controlling for SERT availability in the left amygdala.

**Controlling for the cerebral blood flow**

Formally, in the compartmental model, BP_ND_ (as k_on_.B_max_/k_off_ ratio) is independent of blood flow. So, modeled BP_ND_ should not be altered by increases in cerebral blood flow ^2,3^ and thus parameters related to brain blood transfer constants in the SRTM model (k2’ and R1). To ensure this independence between BP_ND_ and cerebral blood flow, we first extracted clearance kinetic parameters (k2') from the DRN and performed correlations between these parameters and BOLD activation of the amygdala. The results did not show significant correlation between the k2' in the DRN and the BOLD signal related to the activity of the amygdala (Pearson correlation, p=0.267 and p=0.118 for the left and right amygdala respectively). Thus, it appears that the clearance of the tracer in the DRN does not alter BP_ND_ estimation and is not driving the observed effect. In the same way, we extracted the values of R1 (that represent the variations in relative perfusion between target and reference regions, K_1tgt_/K_1ref_) and conducted the same correlation analysis. The results show a significant correlation between the R1 of the DRN and the BOLD signal of the right amygdala (p=0.023), but not with the BOLD signal of the left amygdala. A correlation analysis between the right amygdala BOLD signal and DRN BP_ND_, controlling for the DRN parameter R1, confirmed the negative correlation observed between DRN BP_ND_ and right amygdala activity (p=0.037, r = -0.390). This suggests that although the BP_ND_ signal is partly affected by changes in relative perfusion between the target (DRN) and the reference (cerebellum) regions, the observed correlations appear to be independent of these changes.

The binding in the reference region and its kinetics may affect the outcome of BP_ND_ in the target region, in this case, the DRN. In turn, the binding in the reference region could drive our effect. The relationship observed between the BOLD signal of the amygdala and the BP_ND_ in the DRN could result from this variation. To confirm that it is not the case, we extracted the time-activity-curves (TACs) of the cerebellum and expressed them in standardized uptake value (SUV, see **supplementary figure S3**). We then computed the area under the TACs. To this end, we performed numerical integration *via* the trapezoidal method implemented in MATLAB for each section of the curve defined by two time points. Summing these integrals gave us an approximation of the area under the curve. We then, correlated these values with the BOLD signal related to the activity of the amygdala to tackle the possibility that the binding of the reference region could drive our main relationship. Pearson correlation revealed no significant link between the area under TACs and the BOLD signal related to the left (p=0.833) or right amygdala (p=0.882). We demonstrate that BP_ND_ variations in the target regions and its correlations with BOLD signal are not explained by variations in the reference region (that could be induced by blood flow), confirming that the BOLD signal in the amygdala covaries with the BP_ND_ of the SERT in the DRN.

We also confirmed that the relationship between the BP_ND_ in the DRN and the prefronto-amygdala connectivity is independent of both the blood flow and the transfer parameters. To achieve this, we performed a similar gPPI analysis using the R1, k2, and the area under the time-activity-curve parameters instead of the BP_ND_ in the DRN. Using each parameter, we looked for any covariation in the connectivity according to the parameter. None of these analyses revealed significant connectivity modulation according to the parameter when controlling for the FWE in the brain. Finally, using the spherical ROI of the ACC, we conducted a small volume correction with each of our variables as covariate. Here again, no significant correlations were observed. This confirms that the connectivity modulation observed between the amygdala and the dlPFC/ACC is independent of the kinetic estimation of blood flow in the brain.

**Effect of the DRN SERT availability on the functional connectivity of a prefronto-amygdala circuit when viewing happy facial emotions**

Results for the left lateral, left medial, and right lateral amygdala revealed no significant influence of SERT availability in the dorsal raphe nucleus (DRN) on functional connectivity (even after applying the small volume correction). However, results for the right medial amygdala revealed a significant negative correlation between SERT availability in the DRN and the connectivity with the ACC (x,y,z = 6,36,-4, t = 4.09). Contrary to when viewing negative emotions, the functional connectivity between the amygdala and the ACC covaried according to the level of SERT in the DRN when participants viewed happy faces.

**Supplementary figures**

**
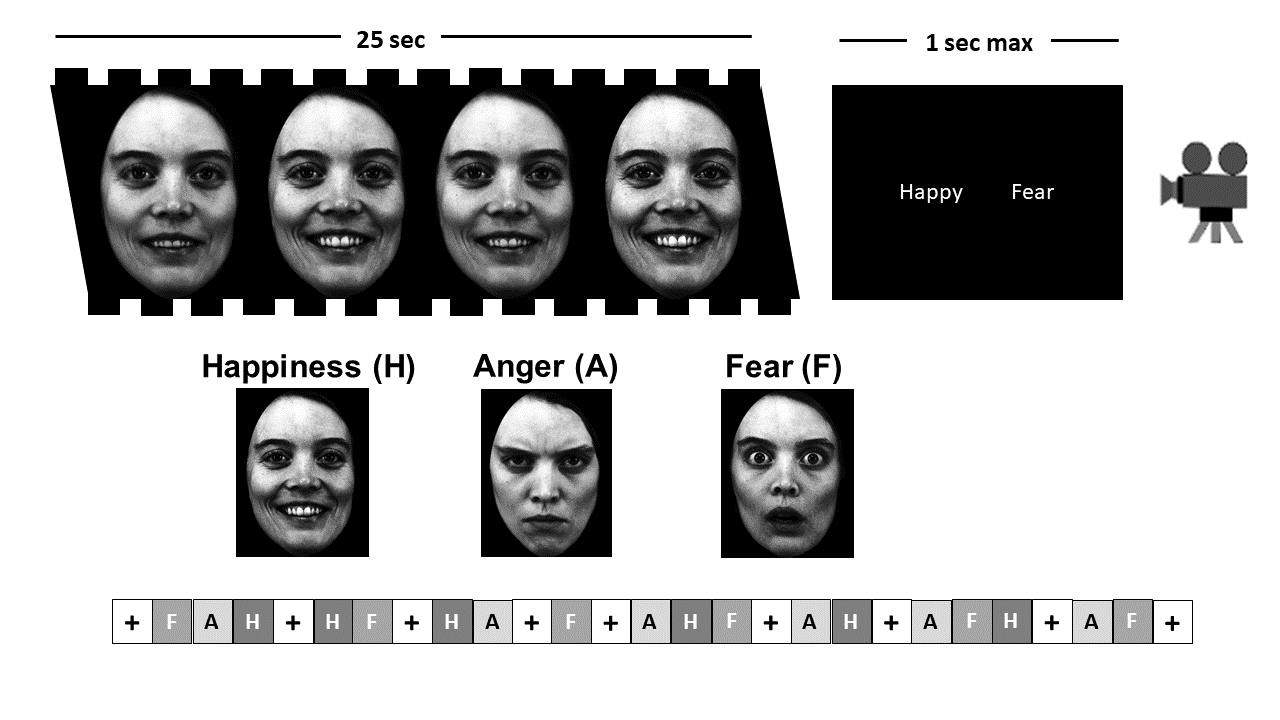
**

**Supplementary figure 1. Dynamic Facial Expression Task design:** each movie clip lasted for 25 seconds and consisted of 50 sequences of faces morphing progressively from neutrality to emotional faces. Then, the subject had one second to identify which emotion he had been shown between two propositions. Three different emotions were presented (Fear, Anger, and Happiness) interleaved by a fixation cross condition as control. Face stimuli originate from the valid and reliable pictures of facial affect ^2^. At the bottom of the figure is an example of a sequence presented to participants.

**Supplementary figure 2. Mean regional non-displaceable binding potential.** Illustration purposes (error bars are SEM). N. accumbens = Nucleus accumbens. L = left hemisphere. R = right hemisphere. PFC = prefrontal cortex. DRN = dorsal raphe nucleus.


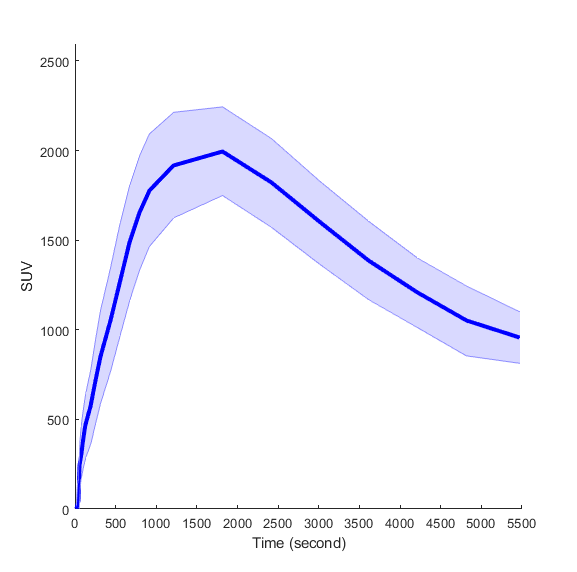


**Supplementary figure 3.** Cerebellum time-activity-curve (TAC) expressed as standardized uptake value (SVU) through time. The SUV of the cerebellum was extracted through time for each participant. The line represents the mean TAC computed across all the participants’ data. The blue shaded area represents the standard deviation.

|  |  |  | **Fix** | **Fear** | **Angry** |
| --- | --- | --- | --- | --- | --- |
| **Left** | **Medial** | **Fix** | - |  |  |
|  |  | **Fear** | 0.001 ** | - |  |
|  | **Amygdala** | **Angry** | <0.001 *** | 1.000 | - |
|  |  | **Happy** | 0.004 ** | 1.000 | 1.000 |
|  | **Lateral** | **Fix** | - |  |  |
|  |  | **Fear** | 0.001** | - |  |
|  | **Amygdala** | **Angry** | 0.001 ** | 1.000 | - |
|  |  | **Happy** | 0.001 * | 1.000 | 1.000 |
| **Right** | **Medial** | **Fix** | - |  |  |
|  |  | **Fear** | <0.001 *** | - |  |
|  | **Amygdala** | **Angry** | <0.001 *** | 1.000 | - |
|  |  | **Happy** | 0.003 ** | 0.540 | 0.004 ** |
|  | **Lateral** | **Fix** | - |  |  |
|  |  | **Fear** | 0.001 ** | - |  |
|  | **Amygdala** | **Angry** | <0.001 *** | 1.000 | - |
|  |  | **Happy** | 0.006 ** | 1.000 | 0.414 |

**Supplementary table 1. Summary of the statistics from *post-hoc* comparisons.** For the left medial as well as for the right lateral amygdala, we performed a repeated measure ANOVA. *Post-hoc* comparisons were conducted only if the repeated measure ANOVA revealed a significant effect of the conditions. Concerning the right medial and the left lateral amygdala, we conducted a Friedman test because the normality assumption was violated. Results of the Friedman tests were not significant.

|  |  |  | **Fix** | **Fear** | **Angry** |
| --- | --- | --- | --- | --- | --- |
| **Left** | **Medial** | **Fix** | - |  |  |
|  |  | **Fear** | 0.001 * | - |  |
|  | **Amygdala** | **Angry** | <0.001 ** | 1 | - |
|  |  | **Happy** | 0.004 | 1 | 1 |
|  | **Lateral** | **Fix** | - |  |  |
|  |  | **Fear** | 0.001* | - |  |
|  | **Amygdala** | **Angry** | <0.001 ** | 1 | - |
|  |  | **Happy** | 0.001 * | 1 | 1 |
| **Right** | **Medial** | **Fix** | - |  |  |
|  |  | **Fear** | <0.001 ** | - |  |
|  | **Amygdala** | **Angry** | <0.001 ** | 1 | - |
|  |  | **Happy** | 0.003 | 0.54 | 0.004 |
|  | **Lateral** | **Fix** | - |  |  |
|  |  | **Fear** | 0.001* | - |  |
|  | **Amygdala** | **Angry** | <0.001 ** | 1 | - |
|  |  | **Happy** | 0.006 | 1 | 0.414 |

**Supplementary table 2. Summary of the statistics from *post-hoc* comparisons.** Same results as in the supplementary table 1, except that the Bonferroni correction considers the number of ROIs and Emotion (n=16).

| **Supplementary table 3. Negative correlation of SERT availability in the DRN and the strength of connectivity between amygdala and prefrontal cortex.** | | | | | | |
| --- | --- | --- | --- | --- | --- | --- |
|  | Seed | | Peak regions | p-val unc. | p-val FWE | Tvalue |
|  |  |  |  |  |  |  |
| gPPI analysis | *Left amygdala* | Medial | 8 40 6 | SVC | 0.015 ^SVC^ | 4.38 |
|  |  | Lateral | No significant regions | | | |
|  | *Right amygdala* | Medial | -40 24 26 | SVC | 0.007 ^SVC^ | 4.74 |
|  |  | Lateral | No significant regions | | | |

**Supplementary table 3: Summary of the effect of SERT availability in the DRN** **on the amygdala-prefrontal connectivity strength, obtained with gPPI analysis.** We observed a significant modulation of the cortical-subcortical connectivity by SERT availability in the DRN only for the right and left medial amygdala. Significant decreases of the connectivity between the right medial amygdala and the left dorsolateral frontal gyrus (x,y,z = -40,24,26; t = 4.74) as SERT availability level in the DRN increased, when viewing negative emotion stimuli compared to fixation (P < 0.05, family-wise error [FWE] small volume corrected within a sphere of radius of 10 mm centered on Neurosynth meta-analysis peak x,y,z = -44,22,24). Similarly, we observed a significant decrease of the connectivity between the left medial amygdala and the anterior cingulate cortex (ACC) (x,y,z = 8,40,6; t = 4.38) (P < 0.05, family-wise error [FWE] small volume corrected within a sphere of radius 10mm centered on meta-analysis peak x,y,z = 0,40,0). Small volume correction (SVC).

**Supplementary references**

1. Sato, W., Kochiyama, T., Yoshikawa, S., Naito, E. & Matsumura, M. Enhanced neural activity in response to dynamic facial expressions of emotion: An fMRI study. *Cognitive Brain Research* **20**, 81–91 (2004).

2. Ekman, P., and Friesen, W. V. (1976). Pictures of Facial Affect. Palo Alto, CA: Consulting Psychologists Press.

3. Salinas, C. A., Searle, G. E. & Gunn, R. N. The simplified reference tissue model: Model assumption violations and their impact on binding potential. *Journal of Cerebral Blood Flow and Metabolism* **35**, 304–311 (2015).

4. Sander, C. Y. *et al.* Effects of flow changes on radiotracer binding: Simultaneous measurement of neuroreceptor binding and cerebral blood flow modulation. *Journal of Cerebral Blood Flow and Metabolism* **39**, 131–146 (2019).
